# Progress of Surgery

**Published:** 1903-08-01

**Authors:** 


### Progress of Surgery.

*(Concluded from page 299.)*

**Rupture of the Bladder.**—An interesting example of this injury has been recorded by Drs. Daly and Harrison of Hull.[4] A man of 36 was admitted to Hull Royal Infirmary with the history that he had been seized with a sudden severe pain in the abdomen after carrying a pail of water. The pain passed into the penis. There was abdominal tenderness and a little dulness in each flank. Ten ounces of clear urine were drawn from the bladder with a catheter. On the following day he became seriously ill with anxious expression, small pulse, shallow respirations, severe abdominal pain and vomiting. Operation refused at first, was performed 64 hours after the onset of symptoms. A large quantity of turbid fluid with a urinous odour was evacuated but there was no peritonitis. A rent was found in the posterior surface of the bladder of sufficient size to admit the tip of the index finger. This was sutured with two rows of Lembert's sutures. The abdomen was washed out. Transfusion was performed during the operation and some adrenalin put into the saline fluid used. Ten ounces of urine were drawn off twenty minutes after the conclusion of the operation. This rapid secretion was probably due to the adrenalin. During the first few days the patient became uræmic and developed also signs of consolidation at the base of the left lung. On the eighth day he ripped open the abdominal wound and pulled out two feet of intestine. This was cleansed and returned to the abdomen and the peritoneal cavity flushed. Slow but steady recovery followed. The writers quote the statistics of Alexander, who collected all the cases which had been treated by suture and recorded up to 1901. The cases numbered 45, of which 23 died and 22 recovered. MacCormac had the first two successful cases in 1886. The diagnosis was difficult in the case recorded above for there was no history of violence, no stricture, and no hæmaturia. In only five recorded cases has the time elapsing between the rupture and the operation been as long as in this case, and of the five only one recovered.

**The Treatment of Stricture of the Urethra.**—This was the subject of special discussion at a recent meeting of the New York Medical Association, and we give an abstract of the views of the leading speakers.[5] J. W. S. Gouley laid down the following general principles with regard to dilatation:—(1) Strictures at the meatus require incision; (2) strictures in the scrotal region which are narrow, indurated, resilient, and refractory require internal incision; (3) strictures which render catheterism difficult or dangerous ordinarily require internal incision if in the scrotal region, and external incision if in the perineal region; (4) strictures in the perineal region which are narrow and refractory to divulsion, or are complicated with fistulæ and accompanied by masses, or are associated with extravasation or abscess formation, all require external incision; (5) traumatic strictures in the perineal region require early external incision; (6) other strictures are generally amenable to dilatation, or at least to divulsion. Gradual dilatation was indicated in the formative stages of stricture, or when the condition of the upper urinary organs would render an operation dangerous. Continuous dilatation was indicated in narrow strictures which did not yield to ordinary dilatation and threatened to cause retention of urine. This method was only a preparatory step to other operative measures, and should not be employed longer than three days. L. W. Hotchkin discussed internal urethrotomy. He said that strictures near the meatus should be divided on the floor of the canal by a bistoury, under the guidance of light as well as touch. The Maisonneuve urethrotome was specially suitable for the narrow strictures which would admit only of the passage of a filiform guide. The Otis urethrotome was useful in strictures of a larger calibre. Parker Syms discussed external urethrotomy. After that operation had been performed the meatus should be divided to its full size, and then with the urethrotome all anterior strictures should be cut. After this perineal drainage for five or six days was recommended, then a full-sized sound should be passed at intervals which were gradually lengthened. F. C. Valentine was strongly of opinion that strictures were better treated by dilatation than by cutting operations. C. B. Lockwood[6] has contributed an article on the technique of internal urethrotomy, and described a urethrotome he has devised for the purpose. The less the urethra was touched before the operation the better the after progress. The writer states that he has no faith whatever in dilatation as a measure of permanent relief. The dangers of septic urine are emphasised. The pubes and scrotum are to be thoroughly cleaned and shaved and the field of operation prepared exactly as it would be for any other aseptic operation. Biniodide of mercury, 1 in 500 in methylated spirit, is used to disinfect the skin of the patient and hands of the surgeon. The urethrotome the writer has had constructed is of small calibre, and cuts accurately from behind forwards, combining the advantages of Teevan's and Thompson's instruments. To make sure of a complete division of the stricture Otis's dilating urethrotome is afterwards used and then a 28 m.m. bougie is passed. An antiseptic dressing is applied over the penis. A bougie is passed for the first time on the tenth day.

[4] Brit. Med. Jour., Jan. 10, 1903, p 76. [5] Med. Rec., Jan. 3, 1903, p. 36. [6] Lancet, Jan. 7, 1903, p. 123.

